# Treatment with CFTR modulators and subsequent remission of AA amyloidosis

**DOI:** 10.1093/ckj/sfag266

**Published:** 2026-08-18

**Authors:** Rachel McDougall, Christopher Hill

**Affiliations:** Regional Centre for Nephrology and Transplantation, Belfast City Hospital, Belfast, UK; Renal, Belfast, UK, Northern Ireland Medical and Dental Training Agency, Belfast, United Kingdom; Regional Centre for Nephrology and Transplantation, Belfast City Hospital, Belfast, UK

**Keywords:** amyloidosis, CFTR, cystic fibrosis, nephrotic syndrome, proteinuria

## Abstract

Cystic fibrosis transmembrane conductance regulator (CFTR) modulators have been revolutionary for cystic fibrosis patients. Their potential influence on AA amyloidosis has not been investigated. In AA amyloidosis, patients develop amyloid deposits of serum A amyloid, in the context of a chronic inflammatory state. This case report is of a gentleman with cystic fibrosis and nephrotic range proteinuria secondary to AA amyloidosis. At 5 years following commencement of CFTR modulators, he is in sustained, complete remission with normal urine protein excretion and serum amyloid protein levels within the normal range.

## BACKGROUND

AA amyloidosis develops in response to chronic inflammation. It often affects the kidneys, usually presenting with proteinuria, which requires renin–angiotensin system inhibition. It can be severe enough to cause nephrotic syndrome. As treatments improve for various inflammatory conditions, AA amyloidosis cases have declined [1]. CFTR modulators, such as Ivacaftor/Tezacaftor/Elexacaftor have this potential for cystic fibrosis patients [2]. These medications improve survival outcomes for patients, and reduce the frequency of pulmonary exacerbations requiring treatment with intravenous antibiotics [2, 3]. This case demonstrates a novel benefit—associated reduction of proteinuria in renal AA amyloidosis.

## CASE REPORT

This 24-year-old gentleman was referred to Nephrology in 2019, with worsening proteinuria. He had a past medical history of cystic fibrosis (F508del/Q493X). His rising urine albumin:creatinine ratio (uACR) was identified at Diabetes clinic, with a uACR of 92 mg/mmol in February, then 226 mg/mmol in April.

At his first Renal outpatient appointment, he had normal estimated glomerular filtration rate (eGFR) but his uACR was higher than the upper limit of the albumin assay and his serum albumin was 26 g/l. He was normotensive at clinic with a blood pressure of 127/78 mmHg. He was started on irbesartan and a biopsy was arranged.

The renal biopsy was in keeping with AA amyloidosis: amyloid that stained positive with Congo red stain, and demonstrated apple green birefringence under polarised light. The amyloid stained with serum A amyloid antibodies.

His irbesartan was uptitrated to maximum dose and his Cystic Fibrosis team reviewed him in July 2019. Given his new diagnosis of AA amyloidosis, they planned to treat any infective exacerbations with prolonged courses of antibiotics. They changed his Colobreathe® (inhaled colistimethate sodium) to Cayston® (inhaled aztreonam lysine) as this was thought to contribute to his exacerbations.

This gentleman was referred to the National Amyloidosis Centre in University College London. He did not meet the echo criteria for cardiac amyloidosis and his serum amyloid P component scan demonstrated amyloid deposits in his spleen, kidneys, and adrenal glands. His serum amyloid A protein was 19.7 in August 2019 and 102 in September. His best uACR achieved before CFTR modulator treatment was 259.03 mg/mmol in October 2020.

The patient was commenced on Kaftrio® (Ivacaftor 75 mg/Tezacaftor 50 mg/Elexacaftor 100 mg) and Kalydeco® (Ivacaftor) in December 2020. At review in March 2021, his proteinuria was improving: uACR had fallen to 205 mg/mmol in association with a serum albumin of 29 g/l. He continued to have a normal eGFR. In July 2021, his serum amyloid A protein level was <3.2 mg/l with a C-reactive protein (CRP) of 1.

**Figure 1:**
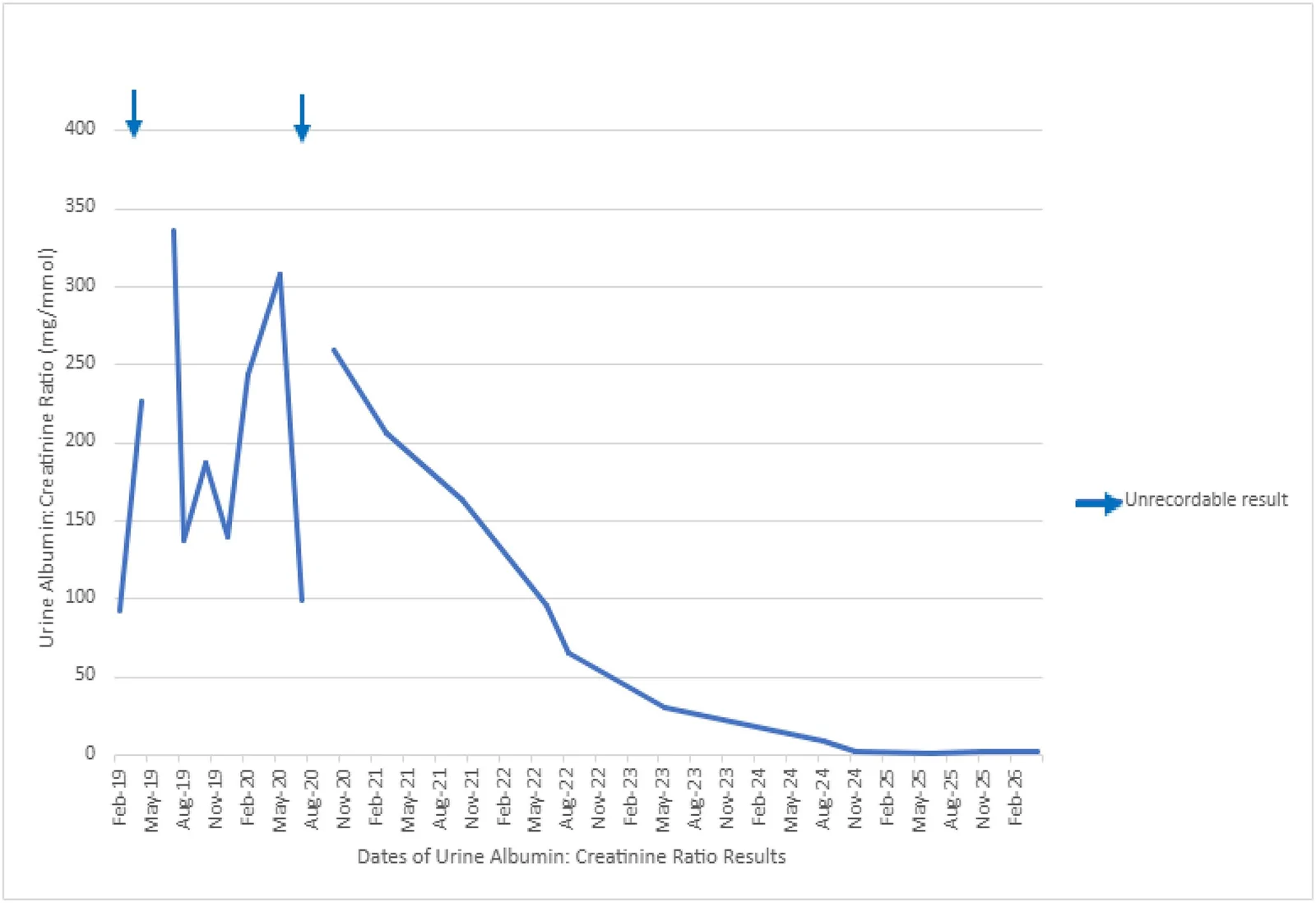
A line graph of the uACR results from February 2019 until April 2026.

There was a progressive fall in uACR over the next 3 years, such that by August 2024 it was 8.9 mg/mmol (see Fig. 1). There were concerns raised about his blood pressure at clinic and in 2024 he had a 24-hour blood pressure monitor, which demonstrated an average blood pressure of 121/75 mHg. By the time of his 2025 renal review, his eGFR was normal with no proteinuria.

## DISCUSSION

This case of a cystic fibrosis patient with AA amyloidosis shows improvement in nephrotic range proteinuria to a uACR within normal range. His uACR had increased to 259.03 mg/mmol despite maximal angiotensin receptor blocker therapy and a satisfactory blood pressure. However, once he started his Ivacaftor/Tezacaftor/Elexacaftor treatment, both his cystic fibrosis symptoms and his proteinuria improved.

CFTR modulators are thought to improve life expectancy of cystic fibrosis patients due to their effect on lung function, but also their reduction of pulmonary exacerbations [2, 3]. This reduction of chronic inflammation will also reduce the incidence of AA amyloidosis. This has been possible to achieve for other chronic inflammatory conditions such as inflammatory arthritides, which has subsequently reduced the number of associated AA amyloidosis cases [1].

This case suggests the same promise for cystic fibrosis-associated AA amyloidosis. Through treatment with CFTR modulators, this patient’s nephrotic range proteinuria has resolved, his renal function has been preserved, and he has remained in remission. In summary, this case highlights additional benefits to CFTR modulator treatment, and the need to treat chronic inflammation sources that drive AA amyloidosis pathogenesis.
